# Adequacy of Hemodialysis Serves as an Independent Predictor of Humoral Response to ChAdOx1 Prime-Boost Vaccination in Hemodialysis Patients

**DOI:** 10.3390/v14061149

**Published:** 2022-05-26

**Authors:** Chun-Yu Chen, Kuan-Ting Liu, Shin-Ru Shih, Jung-Jr Ye, Yih-Ting Chen, Cheng-Kai Hsu, Heng-Chih Pan, Heng-Jung Hsu, Chiao-Yin Sun, Chin-Chan Lee, Chun-Ying Wu, Chi-Chun Lai, I-Wen Wu

**Affiliations:** 1 Department of Nephrology, Chang Gung Memorial Hospital, Keelung 204, Taiwan; shone@cgmh.org.tw (C.-Y.C.); b9402031@cgmh.org.tw (Y.-T.C.); kylegb@cgmh.org.tw (C.-K.H.); balour@cgmh.org.tw (H.-C.P.); r5267@cgmh.org.tw (H.-J.H.); sun3970@cgmh.org.tw (C.-Y.S.); leefang@cgmh.org.tw (C.-C.L.); 2 College of Medicine, Chang Gung University, Taoyuan 333, Taiwan; ccl404@cgmh.org.tw; 3 Research Center for Emerging Viral Infections, College of Medicine, Chang Gung University, Taoyuan 333, Taiwan; jeff31602@gmail.com (K.-T.L.); srshih@mail.cgu.edu.tw (S.-R.S.); 4 Graduate Institute of Biomedical Science, College of Medicine, Chang Gung University, Taoyuan 333, Taiwan; 5 Department of Medical Biotechnology and Laboratory Science, College of Medicine, Chang Gung University, Taoyuan 333, Taiwan; 6 Graduate Institute of Medical Biotechnology and Laboratory Science, College of Medicine, Chang Gung University, Taoyuan 333, Taiwan; 7 Department of Infectious Diseases, Chang Gung Memorial Hospital, Keelung 204, Taiwan; loyalwise@cgmh.org.tw; 8 Department of Laboratory Medicine, Chang Gung Memorial Hospital, Keelung 204, Taiwan; hla0861@cgmh.org.tw; 9 Department of Ophthalmology, Chang Gung Memorial Hospital, Keelung 204, Taiwan

**Keywords:** COVID-19, neutralizing antibodies, vaccine, hemodialysis adequacy, neutralization

## Abstract

Background: Immune response assessed by the quantification of neutralizing antibodies (nAbs) and predictors associated with immunogenicity after the prime-boost ChAdOx1 (Oxford–AstraZeneca) COVID-19 vaccine in hemodialysis (HD) patients remains unclear. Methods: This prospective study enrolled 174 HD patients and 67 healthy subjects to evaluate antibodies against the spike protein 1 and receptor-binding domain of severe acute respiratory syndrome coronavirus type 2 after prime-booster vaccination, by using enzyme-linked immunosorbent assay and applied spline-based generalized additive model regression analysis to predict 50% neutralization titer (NT_50_). The correlation between HD parameters and NT_50_ was analyzed. Results: NT_50_ was lower in HD patients compared with healthy controls after the prime-boost dose (*p* < 0.001). The geometric mean titer ratios were higher in first-dose seronegative than in the seropositive subgroup in HD patients and healthy controls (6.96 vs. 2.36, *p* = 0.002, and 9.28 vs. 1.26, *p* = 0.011, respectively). After two doses of ChAdOx1, one-way ANOVA showed that Ca × P was positively associated with NT_50_ (*p* trend = 0.043) and multiple linear regression showed the similar results (*p* = 0.021). Kt/V (a quantification of dialysis adequacy) (OR = 20.295, *p* = 0.005) could independently predict seroconversion (NT_50_ ≥ 35.13 IU/mL). Conclusion: Adequacy of hemodialysis could independently predict seroconversion in HD subjects vaccinated with prime-boost doses of ChAdOx1.

## 1. Introduction

Severe acute respiratory syndrome coronavirus type 2 (SARS-CoV-2) infection, has been a worldwide pandemic upsetting 442 million people, and continues to provoke a tremendous global burden [1]. Even though vaccination with prime and booster doses were performed in most developed countries, breakthrough infection frequently happened under the ravages of delta and omicron variants [2,3]. Uremic patients are often elderly, concomitant of comorbidities, micro-inflamed and immunocompromised, which might increase susceptibility to coronavirus disease 2019 (COVID-19) [4,5]. In-center hemodialysis (HD) patients usually necessitate permanent attendance at crowded dialysis facilities and often have considerably mitigated immune responses to vaccine. Combining the above unfavorable factors, rapid transmission of COVID-19 in HD facilities had occurred and contributed to a grave mortality in HD patients [6].

The immunogenicity of COVID-19 vaccines in HD cohorts was significantly inferior to the general population, and this under-response was consistent for both mRNA or adenoviral-vector vaccines [7,8,9,10]. For examples, a neutralization assessment in HD patients indicated that a single dose of ChAdOx1 (Oxford–AstraZeneca) would show a 23% positive humoral response rate, and a single dose of mRNA-1273 (Moderna) can result in a 46.2% positive humoral response rate in HD patients. On the other hand, the seroconversion rate was 18–53% after the first dose and 70–96% after the second dose, respectively, in a pooled estimate of healthy controls [7,8]. Measures to increase the immune response of vaccination in HD patients are urgently needed but remains unclear to date.

The COVID-19 vaccines has been proved to be safe and efficacious to prevent severe COVID-19 in HD patients and the inoculation was strongly recommended by the Advisory Committee on Immunization Practices (ACIP) of Taiwan and Taiwan Society of Nephrology [11]. The first large-scale outbreak of COVID-19 has occurred in northern Taiwan since 14 May 2021. At that time, the vaccine was extremely lacking and overall coverage rate of the first dose was only 1.5% in HD patients; consequently, numerous clusters of contraction developed in northern area HD facilities contributed to grave morbidities and mortalities. Furthermore, vaccination has been prioritized for HD patients as well as other immunocompromised cohorts and the ChAdOx1 vaccine was the mandatory first dose option for these patients. Our previous study has demonstrated that cardiothoracic ratio and age would independently predict neutralizing antibody (nAb) titer after a single dose of ChAdOx1 [8]. However, immune responses to repeated doses of vaccine and predictors for low immunogenicity in HD patients are incompletely understood.

Due to the semi-mandatory inoculation policy, the majority of HD subjects have been vaccinated with the second dose of ChAdOx1. To continue tracking the immune response and explore the characteristics of seronegative subgroup, we performed an observation cohort study to compare the titers of nAb between HD patients and healthy controls who have been fully vaccinated with ChAdOx1, and further demarcated the possible dialysis-related factors affecting the production of nAbs. This result may help us to optimize dialysis program and formulate an effective vaccination strategy in HD subjects.

## 2. Materials and Methods

### 2.1. The Study Design and Patient Characteristics

This observational, prospective and single-center study assessed the nAb response in HD patients and healthy controls after the standard prime-booster dose of the ChAdOx1 COVID-19 vaccination implemented in Chang Gung Memorial Hospital Keelung Branch in Taiwan. ChAdOx1 vaccines were uniformly supplied to HD facilities for injection, in accordance with the COVID-19 inoculation policy promulgated by the national health authorities and Taiwan Society of Nephrology. Participants who completed the two doses of vaccination were included to the study. HD patients, who had an active major illness (uncontrolled malignancy, unstable cardiovascular disease, Child–Pugh liver cirrhosis score worse than A), a history of prior infection with SARS-CoV-2 or unwilling to receive repeated doses of ChAdOx1 vaccine or to participate in the assessment were excluded. The eligible HD patients who have symptoms of a cough, sore throat, diarrhea and loss of taste or smell had to undergo a rapid antigen test before enrollment. The healthy control group (normal renal function people) were recruited from the healthcare staff and out-patient clinics of the hospital, in compliance with the same enrollment criteria. SARS-CoV-2 rapid antigen tests were obligatory in all of our HD patients once weekly, and misjudged the false positive humoral response due to previous infection. Due to the shortage of the vaccine, the second dose vaccination was postponed to the 16th week after first dose. The differences of evolution of nAb titers between HD patients and healthy controls were evaluated. In addition, predictors of the immunogenicity to the first dose vaccination between two groups, as well as indicators correlated to the trajectory of nAb titers after two doses of vaccination, were analyzed. This study was performed in accordance with the Declaration of Helsinki and was approved by the Ethics Committee of the Institutional Review Board at Chang Gung Memorial Hospital (IRB: 202001041A3C604 and 202100854B0A3). Written informed consent was obtained from all study participants.

### 2.2. Sample Collection

Blood samples were collected after overnight fasting and were delivered immediately (within 4 h of collection) to the laboratory for biochemical analyses, complete blood counts and antibody titers. Blood samples of HD patients were collected via venous chamber before a single dialysis treatment. A fraction of the samples was relocated to chilled tubes and centrifuged at 3000× *g* for 10 min at 4 °C to obtain the sera. Lipemic or hemolyzed sera were discarded.

### 2.3. Humoral Response Assessment

Humoral response was assessed by measuring nAb response on day 56 after the prime dose and day 35 after the booster dose of ChAdOx1 COVID-19 vaccine in HD patients and on a median of 30 (26–50) days after the prime dose and 21 (17–28) days after the booster dose of ChAdOx1 COVID-19 vaccine in healthy controls, using the Formosa Biomedical Technology MeDiPro SARS-CoV-2 Antibody ELISA assay, which detects antibodies against SARS-CoV-2 viral spike protein 1 (S1) and receptor-binding domain (RBD). MeDiPro is a kit for quantifying neutralizing antibodies, which was technology transferred from Research Center for Emerging Viral Infections, Chang Gung University and has been approved by Taiwan Food and Drug Administration (No. 1106803303). The data for S1 and RBD fusion proteins can precisely predict the SARS-CoV-2 50% neutralization titer (NT_50_) under a two-variable generalized additive model and WHO international unit conversion. The assay has a 92.2% (95% CI, 84.0%–96.4%) sensitivity and 93% (95% CI, 81.4%–97.6%) specificity [12].

### 2.4. Quantifying nAbs by a Two-Variable Generalized Additive Model

In our previous study, we have demonstrated the correlation between the nAb titer and the S1 and RBD antibody responses, respectively, obtained from a pseudotyped SARS-CoV-2 spike lentivirus neutralization test (NT) in biosafety level 3 laboratory [13]. The binding of S1 (R^2^ = 0.830) and the RBD (R^2^ = 0.870) was well correlated with the log-transformed actual NT titer. We further applied a spline-based generalized additive model (GAM) regression analysis to predict NT_50_. The GAM using S1 and the RBD as two variables gets the highest R^2^ value of 0.917, between the binding capacity and actual NT_50_. The MeDiPro SARS-CoV-2 antibody ELISA was technology transferred from the Research Center for Emerging Viral Infections, Chang Gung University, Taiwan. The MeDiPro using enzyme-linked immuno-sorbent assay (ELISA) was developed to disclose SARS-CoV-2 nAbs in the serum, through the binding affinity of S1 and RBD to antibodies. The nAbs mainly bind to the RBD, which has been universally covered by S1. S1 also appears in several other regions and is imperative for nAb binding. The assay conglomerates each of the S1 and RBD ELISA unit (EU) values, and applies a GAM regression analysis (using S1 and RBD as two predictors) to predict NT_50_ by combining multiple smooth functions [13].

### 2.5. WHO International Standard Unit (IU) Conversion

For the purpose of facilitating the conversion of geometric mean titers (GMTs) of NT_50_ to international units, WHO international standard (IS) sera (20/130, 20/136, and 20/268) were obtained from the National Institute for Biological Standards and Control (NIBSC). IS sera were used to obtain nAb titers in IU/mL. The NT_50_ values for WHO IS sera were determined by a live virus microneutralization assay. Each standard serum sample was tested in duplicate, except for 20/130. For the conversion of NT_50_ to IU/mL, a neutralizing assay was designed to calculate the GMTs of the NIBSC serum samples. Values of <12.31 IU/mL (neutralizing titer < 2.56) were defined as a negative humoral response, values between 12.31 and 35.13 IU/mL (2.56 ≤ neutralizing titer < 8) were defined as a weakly positive humoral response, and values > 35.13 IU/mL (neutralizing titer > 8) were defined as a positive humoral response. NT_50_ ≥ 35.13 IU/mL was also defined as seropositive and NT_50_ < 35.13 defined as seronegative. When we enter the value of the ELISA’s limitation of detection (LOD) into the model, a NT_50_ cutoff value of 12.31 IU/mL was obtained. The cutoff value of 35.13 IU/mL was from converting the lowest neutralizing titer to IU/mL. In clinical practice, the serial dilutions of the virus neutralization assay commenced from 1:8, because any lower dilution of serum was toxic to the cells and resulted in bias in determining the titer. When inputting the neutralizing titer = 8 to the model, a value of 35.13 IU/mL is obtained.

### 2.6. Statistical Analysis

Continuous variables were tested for normal distribution using skewness, kurtosis, and the Kolmogorov–Smirnov test. Normally distributed variables, with values expressed as means (standard deviations), were compared by one-way analysis of variance (ANOVA); while categorical variables were tested using the chi-squared test. The nonparametric independent Kruskal–Wallis test was performed to compare the non-normally distributed variables, expressed as medians (interquartile ranges).

Geometric mean fold increase in titers (GMT ratio) was used to compare nAb responses after the prime and booster dose between HD patients and healthy controls. Seroconversion was compared using a Student’s t-test, and humoral response were compared by the nonparametric independent Mann–Whitney U test. Simple linear regression was applied to examine the association between independent variables and NT_50_ after the booster dose of ChAdOx1 vaccination. The non-normally distributed variables were log- transformed as appropriate. Multiple regression was used to adjust all the confounding factors (model 1) or all these factors, excluding anti-S1 and anti-RBD antibodies (model 2). Univariate, followed by multivariable logistic regression analysis (enter method) was used to assess the odds ratio of clinically variables associated with positive humoral response (NT_50_ cutoff value over 35.13 IU/mL) after the second doses of vaccination in those HD patients were formerly seronegative to the prime dose. 

The receiver operating characteristic (ROC) curves were plotted to predict the probability of a binary outcome, including Ca × P vs. positive humoral response and age vs. positive humoral response. Differences were examined using the area under the receiver operating characteristic (AUROC) curve. All statistical analyses were two-tailed, and a value of *p* < 0.05 was considered statistically significant. Data were analyzed using the Statistical Package for the Social Sciences (SPSS, Inc., Chicago, IL, USA) version 26.0 for Mac. GraphPad Prism version 9 (GraphPad Software, Inc., La Jolla, CA, USA) was used to calculate GMTs with 95% confidence intervals (CIs), and to generate all of the graphs.

## 3. Results

### 3.1. Study Design and Subject Characteristics

**Figure 1:**
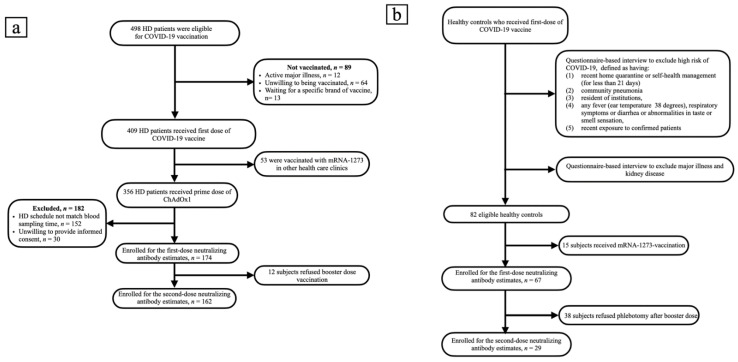
(**a**) Flow chart of patients on hemodialysis selected for neutralizing antibody measurement. (**b**) Flow chart of recruitment of healthy controls.

**Figure 2:**
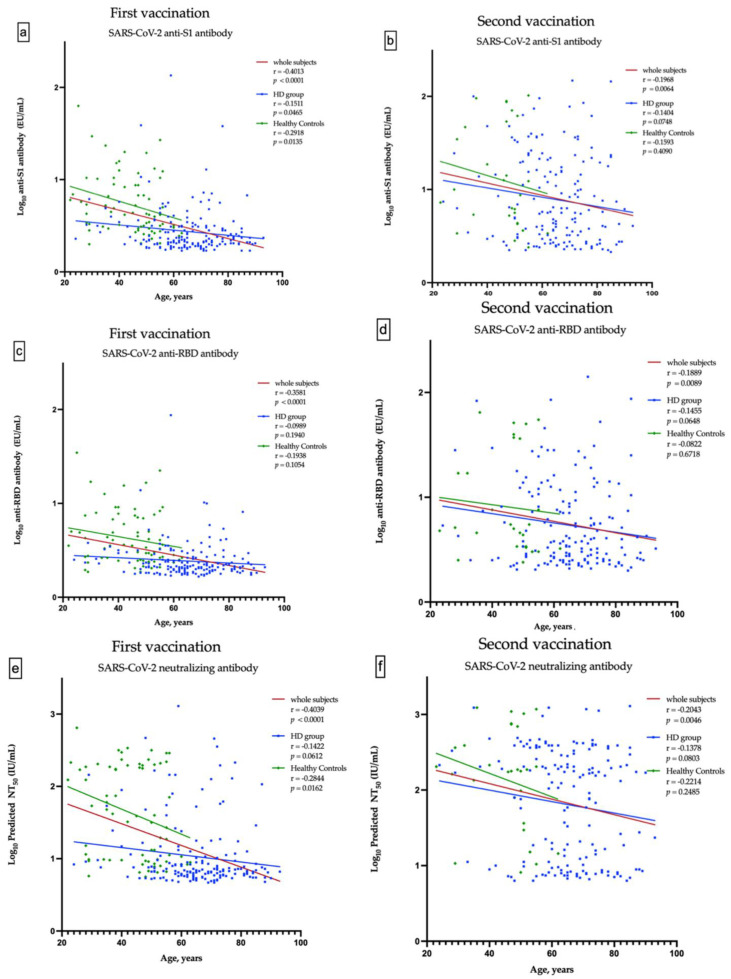
Correlation between the age and anti-S1 antibody after the first and second vaccination (**a**,**b**), between the age and anti-RBD antibody after the first and second vaccination (**c**,**d**), and between the age and predicted SARS-CoV-2 50% neutralization titer (NT_50_) after the first and second vaccination (**e**,**f**).

A total of 498 HD patients were screened. From them, 174 patients, who received the first dose of ChAdOx1, were enrolled into the study. Subsequently, 162 patients completed two doses of vaccination (Figure 1a). The healthy control group consisted of 67 subjects, who received the first dose of ChAdOx1 vaccine. Only 29 of them had the second dose of vaccine and have completed nAbs assessment (Figure 1b). The enrolled fully vaccinated HD subjects had comparable age, gender, albumin, Kt/V, Ca × P product and cardiothoracic ratio to non-enrolled HD subjects (Table S1). The Pearson’s correlation coefficients (r) between age and anti-S1 were −0.1511 and −0.1404 (*p* = 0.0465 and *p* = 0.0748) in the first dose and the prime-boost doses vaccinated HD subjects, respectively (Figure 2a,b); anti-RBD were −0.989 and −0.1455 (*p* = 0.1940 and *p* = 0.0648), respectively (Figure 2c,d); and NT50 were −0.1422 and −0.1378 (*p* = 0.0612 and *p* = 0.0803), respectively (Figure 2e,f). Overall, the values of NT50 appear to be distributed in a descending manner from low age to high age (r = −0.2043, *p* = 0.0046) (Figure 2f). 

### 3.2. Comparison of Demographics and Clinical Characteristics between the Groups with Various Humoral Response to Two Doses of ChAdOx1

HD patients who have received standard prime-booster dose of ChAdOx1 vaccination were stratified according to NT_50_ of nAbs. In parallel with the increase of levels of anti-S1 and anti-RBD antibodies, the values of Ca × P product (*p* trend = 0.043) and P (*p* trend = 0.043) ascended monotonically from the low titer group to high titer group (Table 1). Based on the humoral responses after first and second dose of vaccination, we divided HD patients into three groups: all seronegatives (−/−), first negative but second positive (−/+) or all seropositives (+/+) (Table 2). The dual seropositive patients (group 3) have a higher proportion of cerebrovascular disease (*p* trend = 0.005), a lower proportion of congestive heart failure (p trend = 0.006) and a younger age (*p* trend = 0.04). In addition, cardiothoracic ratio tends to be lower and Ca × P product tends to be high in dual seropositive (*p* trend = 0.071 and p trend = 0.057, respectively) (Table 2).

### 3.3. NT_50_ and Clinical Characteristics of Prime-Boost ChAdOx1 Vaccinated HD Patients

Simple linear regression analysis for factors associated with NT_50_ showed that anti-S1 and anti-RBD antibodies (β ± SE: 1.125 ± 0.209, *p* < 0.001; and β ± SE: 1.448 ± 0.274, *p* < 0.001, respectively) were positively associated with NT_50_. Multiple linear regression analysis with a backward stepwise selection method was performed to assess factors associated with NT_50_ (Table 3). After adjusting all variables (Model 1), time average urea concentration (β ± SE: −0.006 ± 0.002, *p* = 0.008) was significantly and negatively associated with NT_50_, while anti-Si antibodies (β ± SE: 1.396 ± 0.052, *p* < 0.001), hemoglobin (β ± SE: 0.076 ± 0.032, *p* = 0.018), alanine transaminase (β ± SE: 0.287 ± 0.111, *p* = 0.011) and Ca × P product (β ± SE: 0.005 ± 0.002, *p* = 0.007) were positively associated with NT_50_. Although CRP is not statistically significant, it still deserves our attention (β ± SE: −0.079 ± 0.041, *p* = 0.055). Furthermore, multiple linear regression analysis was conducted with all variables, excluding anti-S1 and anti-RBD antibodies (Model 2), and found that Ca × P product (β ± SE: 0.010 ± 0.004, *p* = 0.021) were positively associated with NT_50_, while serum creatinine (β ± SE: −0.062 ± 0.029, *p* = 0.034) were negatively associated with NT_50_. Age tends to be negatively associated with NT_50_ (β ± SE: −0.009 ± 0.005, *p* = 0.079) (Table 3). Figure S1 shows a receiver operating characteristic curve illustrating the performance of Ca × P (AUC: 59%), age (AUC: 43%), and the combination obtained by Ca × P dividing age (AUC: 60%) in predicting the development of nAb titers over 35.13 IU/mL after prime-boost doses of ChAdOx1 vaccination.

### 3.4. Predictors Associated with Seroconversion after Second Vaccination in Previously First-Dose Seronegative HD Patients 

Binary univariate followed by multiple logistic regression analyses were used to appraise the risk of clinical and dialysis-related parameters linked to seroconversion (NT_50_ over 35.13 IU/mL) in the subgroup of previously seronegative (first-dose seronegative) HD patients after the first vaccination (Table 4). The multiple logistic regression (enter method, Model 1) found that male (OR: 0.185, 95% CI: 0.048–0.709, *p* = 0.014) was independently negatively related to seroconversion and Kt/V (OR: 20.295, 95% CI: 2.486–165.683, *p* = 0.005) was independently positively linked to seroconversion after adjusting all variables. Model 2 multiple logistic regression (backward: Wald method) shows similar results (Kt/V, OR: 7.469, 95% CI: 1.283–43.484, *p* = 0.025). The results suggested that adequacy of dialysis might contribute to immunogenicity in first-dose seronegative subjects.

### 3.5. Differences of Humoral Responses between HD Patients and Healthy Controls

**Figure 3:**
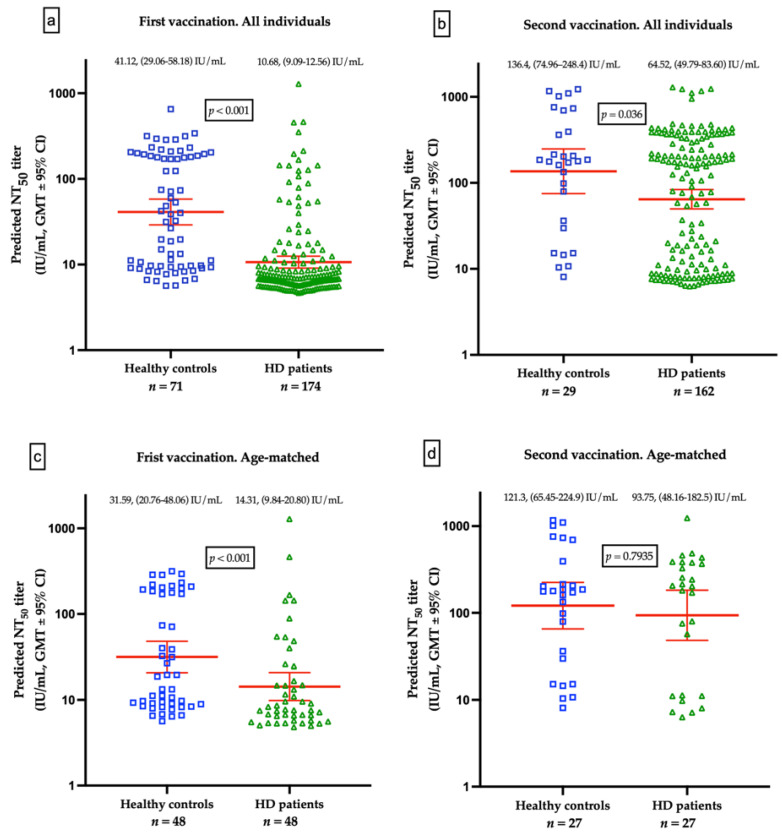
(**a**) Comparison of geometric mean titers (GMTs) for neutralizing antibodies (nAbs) between healthy controls and hemodialysis (HD) patients vaccinated with a first dose of ChAdOx1. (**b**) Comparison of GMTs for nAbs between healthy controls and HD patients after prime-booster doses of ChAdOx1. (**c**) Comparison of GMTs for nAbs from age-matched healthy controls and HD patients vaccinated with a first dose of ChAdOx1. (**d**) Comparison of GMTs for nAbs from age-matched healthy controls and HD patients after prime-booster doses of ChAdOx1.

**Figure 4:**
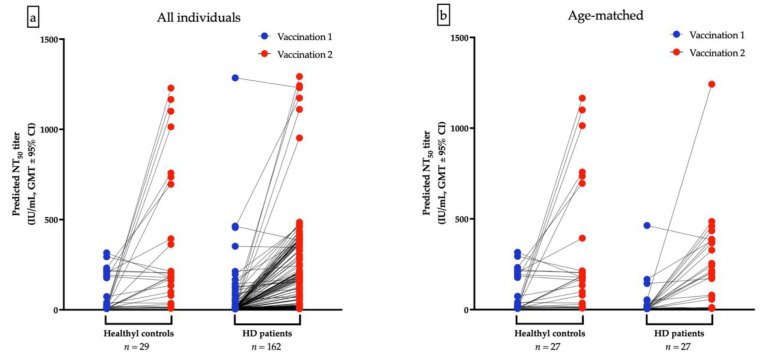
Individual NT_50_ courses in all and in age-matched individuals after the first and after the second ChAdOx1 vaccination are illustrated (**a**,**b**), respectively.

The GMTs for nAbs of ChAdOx1-vaccinated were lower in HD patients than the healthy control after the first dose (10.68 IU/mL vs. 41.12 IU/mL, *p* < 0.001, Figure 3a) and after second dose of ChAdOx1-vaccination (64.52 IU/mL vs. 136.4 IU/mL, *p* = 0.036), respectively (Figure 3b). Due to the significant variance of age between HD and healthy controls, a resampling subset of patients with individual match in age (±2 years) was built. Again, we found that healthy controls had higher GMTs for nAbs than HD patients after receiving the first dose of ChAdOx1 (31.59 vs. 14.31 IU/mL, *p* < 0.001). However, the GMTs after the second dose were not significantly different between the two groups (121.3 vs. 93.75 IU/mL, *p* = 0.7935, Figure 3c,d). The discrepancy may attribute in part to the relatively small sample size and shorter interval between blood sampling and the second dose in healthy controls. Individual NT_50_ courses in all and in age-matched individuals after the first and after the second ChAdOx1 vaccination are illustrated in Figure 4a,b, respectively. The seroconversion rate of HD patients and healthy controls are 13.2% vs. 58.0% (*p* < 0.001) and 50.7% vs. 75.9% (*p* < 0.001) after the first dose and after the second dose, respectively (Table 5). GMT ratio of HD patients is non-inferior to that of healthy controls (geometric mean: 6.01, 95% CI: 4.72–7.66 vs. geometric mean 4.35, 95% CI: 2.14–8.82, *p* = 0.135) (Table 5). Specifically, the finding suggested that although attenuation of humoral response after a single dose in uremic patients, the potential soaring NT_50_ after the second dose in HD patients was noteworthy, especially in younger subjects.

### 3.6. Evolution of nAbs Amount Analyzed by Whether Seroconversion or Not after the Prime Dose

**Figure 5:**
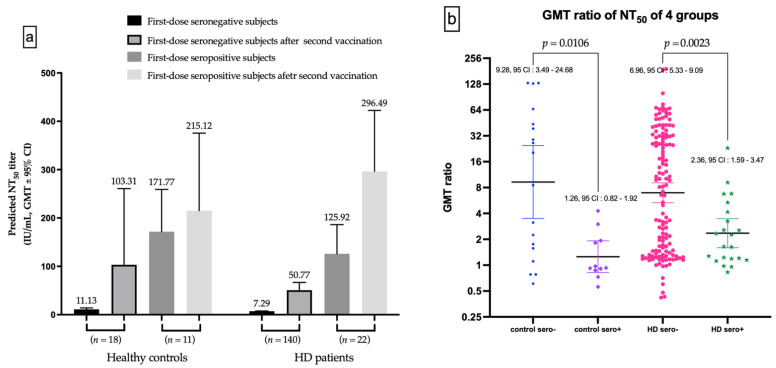
(**a**) The Difference of NT_50_ evolutions in first-dose seronegative and first-dose seropositive subgroup. (**b**) The difference of gemetric mean titer (GMT) ratio between first-dose seronegative and first-dose seropositive controls, and between first-dose seronegative and first-dose seropositive hemodialysis (HD) patients.

The seronegative subgroup after the first dose vaccination has substantial increased in GMT for nAbs after the second vaccination, in both healthy controls and HD subjects (Figure 5a). The GMT ratio of first-dose seronegative controls was higher than first-dose seropositive controls (9.289 vs. 1.255, *p* = 0.0106), as well as for HD subjects (6.964 vs. 2.355, *p* = 0.0023, Figure 5b).

## 4. Discussion

In this prospective observational single-center study, we demonstrated that dialysis adequacy (Kt/V) can independently predict seroconversion after two doses of the ChAdOx1 prime-booster vaccination in first-dose seronegative HD patients. The relationship remained significant after adjusting for albumin, gender, diabetes and immunosuppressant usage. The seroconversion rate of HD patients was lower than that of healthy controls. Furthermore, the GMT ratio for nAbs of the first-dose seronegative subgroup was significantly higher than that of the seropositive subgroup, in both HD subjects and healthy controls.

Previous studies have showed that mRNA vaccines could elicit a more prominent humoral response than the vector-based vaccines in dialysis cohorts, and the humoral response wanes over time according to initial antibody titers elicited by different types of vaccine [8,14,15,16,17]. Although dialysis patients were also proved to generate attenuated nAbs compared with general population after COVID-19 vaccination, full vaccination with prime-booster doses can also provide protection against infection, severe disease, hospitalization and mortality [8,18,19,20]. Especially, El Karoui et al. found a lower relative incidence for dialysis patients, suggesting an effect of vaccination coverages and preventive measures [21]. The results of our study have confirmed a substantial increase in nAbs titer in immunocompromised HD subjects after the second dose.

Several investigations evaluated humoral responses of vaccination, by estimating the anti-S1 or anti-RBD IgG or IgM as a surrogate for nAbs; however, the exact viral neutralization test in uremic cohort remains limited. A UK study used live virus neutralization in seronegative HD patients to compare the humoral response of ChAdOx1 (n = 53) and BNT162b2 (n = 55). They found that the mRNA vaccine provokes comparable nAb titers between HD subjects and controls; nevertheless, the ChAdOx1 elicited lower nAb quantity in HD patients against all variants of concern [15]. Our study identified the concentration of both anti-S1 and anti-RBD antibodies including IgG, IgM and IgA by ELISA procedure and the results were computed by a spline-based two-variable GAM to predict real NT_50_ for nAbs. The robustness of the correlation between predicted and actual virus neutralization titers was validated, which was as high as 0.917 [13], and was superior to many widely used commercial assays, such as Roche and Abbott RBD antibody titers [12]. To our knowledge, this study is the first attempt to explore NT_50_ in an extensive HD cohort inoculated with prime-booster ChAdOx1 using the International Unit. 

High Ca × P product had been proposed as a predictor of soft tissue or vascular calcification, and mortality. A target below 55 has been recommended by the 2003 National Kidney Foundation Kidney Disease Outcomes Quality Initiative (K/DOQI) practice guideline [22]. However, the clinical value of Ca × P product has been controversial and an updated 2009 Kidney Disease: Improving Global Outcomes (KDIGO) clinical practice guideline has suggested to evaluate serum Ca and P individually, instead of tracking Ca × P [23,24]. Our study results indicated Ca × P was significantly positively associated with NT_50_ in both multiple linear regression and one-way ANOVA analyses. The explanation to this phenomenon remains unclear, however, the best cut-off value to induce an appropriate humoral response was 42.92, below the value recommended by the K/DOQI. The Ca^2+^ plays crucial role in the signaling of lymphocytes; the P was considered as part of an integrated approach to support immune functions and sustain a steady microbial ecosystem in the gut; and the Ca × P probably had mutual influence on the regulation of the immune system [25,26]. Further animal experiments are needed to elucidate the relationship between immunogenicity to COVID vaccine and Ca × P in HD patients.

The Kt/V has been used as an indicator of the quantification of dialysis adequacy and was derived by applying the following formula: dialysis clearance of urea (K) multiplied by dialysis time (t), divided by the volume of distribution of urea (V). The delivered dose of dialysis could be adjusted by dialyzer, dialysis time, dialysate flow, blood flow and patient body size. Kt/V has become the preferred method for estimating a delivered dialysis dose because it adequately reflects urea removal and also can be applied to modify the dialysis prescription for those still having residual renal function. The National Cooperative Dialysis Study (NCDS) have demonstrated a strong effect of Kt/V on short-term outcome and led to the widespread deliberation of Kt/V as a standard for evaluating dialysis adequacy. A minimal single-pool Kt/V of 1.2 was developed on the basis of the NCDS and another observational data set, while a Kt/V of lower than 0.8 related to a worse outcome [27,28]. The Hemodialysis (HEMO) Study confirmed that a dose of dialysis significantly greater (Kt/V of 1.71) than Kt/V of 1.2 failed to offer extra survival benefit in HD patients [29]. Our study showed that the quality of the HD assessed by the Kt/V was linked to a seroconversion after the second vaccination, suggesting that uremic toxin plays a crucial role in the immunogenicity after vaccination, especially in previous seronegative HD patients [30]. The phenomenon was also observed in virus-unexposed non-immunosuppressed HD cohorts [9]. In the uremic milieu, defects of innate and adaptive immunity were found with antigen presenting cell dysfunction and attenuated antibody production by damaged differentiated B cell [31,32,33,34]. The impairment of the antigen-presenting capability of the immune system of uremic patients can impede recognition of pathogens and interfere with production of downstream adaptive immunity [35,36]. It has been suggested that end-stage renal disease may be associated with an impaired antibody production post vaccination. In fact, lower Kt/V has been associated with tempered humoral response to hepatitis B vaccine in HD patients [37,38]. Therefore, it is reasonable to conclude that enhancing uremic toxin removal could improve immunogenicity after vaccination.

Albumin, C-reactive protein, transferrin saturation and normalized protein catabolic rate were imperative indicators of clinical outcome in HD patients; however, these parameters were not correlated with levels of NT_50_ in our study. The findings were consistent with a study from Mexico’s healthcare workers, indicating that gender and comorbidities (such as diabetes, obesity and hypertension) have not been linked to low titers of nAbs. A relative homogenous status regarding nutrition, inflammation and administered dialysis dosage might also show this [39,40].

The GMT ratios were calculated from titers after the booster dose, dividing titers after the prime dose. We observed that HD patients have a comparable GMT ratio to that of healthy controls, while the Folegatti et al. also found the similar GMT ratio of approximate 4-fold after prime-boost ChAdOx1 vaccination [41]. Rincon-Arevalo et al. have demonstrated that the responses of B and plasma cells were delayed 3–4 weeks post a booster dose of an mRNA COVID-19 vaccine [42]. Here, our first-dose seronegative HD and healthy controls both had significantly higher GMT ratios than those of first-dose seropositive subjects. This suggests that the first-dose seronegative individuals might have delayed humoral response that could be immensely stimulated by a boost dose. Our findings suggest that full prime-boost vaccination is necessary, especially in immunocompromised individuals such as HD patients. 

This study has several limitations. First, we did not estimate nAbs before vaccination and the presence of a previous occult infection was not clear. However, there was almost no domestic transmission before May 2021 in Taiwan, and there was no laboratory confirmed cases in our HD facility in the period of research. The preventive measure of our facility has included SARS-CoV-2 rapid antigen testing on a weekly basis and may have avoided the presence of asymptomatic infection in our HD patients [43]. Second, the clinical characteristics of healthy controls in this single-center study was lacking and the HD patients and healthy controls are heterogenous in the timing of nAbs assessment. For the convenience of HD patients and to improve compliance with the study, the timing of nAbs measurement must match their monthly scheduled phlebotomy. Third, we did not perform baseline laboratory testing for healthy controls, because they were generally fair, and we simply obtained blood samples for measurement of nAbs. Fourth, the case numbers of healthy controls were less than the HD patients and the loss of a substantial number of healthy controls to obtain blood samples after booster dose might affect the final statistics of comparing with HD patients. Immunogenicity of COVID-19 vaccination in the general population has been extensively investigated in the literature. For this reason, we have emphasized immune reaction in HD patients. The timing and vaccine resource were subjects to national public health policies and could not be controlled even in academia. We have employed a resampling subset of patients, in terms of age, to minimize possible confounding factors as much as possible. Finally, we did not obtain NT_50_ titers from real neutralization testing; however, our methodology has been proven to be superior to other commercial serological tests [12]. Further, a large multi-ethnic cohort is needed to validate the results of present study.

## 5. Conclusions

This prospective study has established the association between hemodialysis adequacy and positive humoral response in a first-dose seronegative HD cohort vaccinated with prime-boost doses of ChAdOx1 COVID-19 vaccine. Significant increases of GMT ratios were seen after booster dose in first-dose seronegative patients. Ca × P product was significantly associated with NT_50_. We also noticed that higher Kt/V was associated with a positive immune response of the boost dose of vaccination in patients who were previously first-dose seronegative. The results of the present study suggest that double dose vaccination is necessary in HD cohorts and pursuit of appropriate dialysis adequacy might contribute to better humoral response post vaccination.

## Figures and Tables

**Table 1:** Comparison of demographics and clinical characteristics between the groups with various humoral responses after two doses of ChAdOx1.

	Negative NT_50_ < 12.31 IU/mL (n= 49)	Weakly Positive 12.31 ≤ NT_50_ < 35.13 (n = 19)	Positive NT_50_ ≥ 35.13 (n = 94)	*p* for Trend
Age, year	65.67 ± 13.27	70.84 ± 10.46	63.33 ± 13.33	0.215
Male, n (%)	27 (55.1)	8 (42.1)	54 (57.4)	0.472
Comorbidities, n (%)				
Diabetes	26 (53.1)	11 (57.9)	48 (51.1)	0.778
Dyslipidemia	15 (30.6)	7 (36.8)	42 (44.7)	0.100
Liver cirrhosis	1 (2.1)	1 (5.3)	2 (2.1)	0.936
Cardiovascular disease	18 (36.7)	5 (26.3)	32 (34)	0.814
Baseline medications, n (%)				
Immunosuppressant	4 (8.2)	0 (0)	5 (5.3)	0.568
RAAS blockade	26 (53.1)	8 (42.1)	38 (40.4)	0.160
β-blocker	25 (51)	8 (42.1)	33 (35.1)	0.066
Statins	13 (26.5)	7 (36.8)	40 (42.6)	0.062
Anti-S1 Abs (EU/mL)	2.62 (2.45–2.91)	3.90 (3.60–4.09)	15.10 (7.22–29.35)	<0.001 *
Anti-RBD Abs (EU/mL)	2.40 (2.24–2.56)	2.97 (2.87–3.24)	7.52 (4.26–14.67)	<0.001 *
Hemoglobin (g/dL)	9.93 ± 1.23	10.11 ± 1.10	10.20 ± 1.18	0.207
WBC (1000/μL)	6.00 (4.60–7.80)	6.80 (4.60–7.80)	6.00 (5.00–7.60)	0.438
Platelet (1000/μL)	189.20 ± 71.37	164.89 ± 56.89	199.96 ± 68.94	0.274
Albumin (g/dL)	4.02 ± 0.41	4.17 ± 0.28	4.03 ± 0.35	0.954
Cholesterol (mg/dL)	156.10 ± 41.45	162.58 ± 41.04	151.59 ± 33.95	0.428
Triglyceride (mg/dL)	144.0 (81.5–211.5)	122 (63–172)	113.00 (80.00–162.75)	0.249
AST (U/L)	16 (13–19)	18 (13–24)	17.00 (13.25–20.75)	0.296
Total bilirubin (mg/dL)	0.4 (0.3–0.4)	0.4 (0.2–0.4)	0.4 (0.3–0.4)	0.848
Bun (mg/dL)	68.99 ± 21,78	76.02 ± 26.99	67.98 ± 19.97	0.666
Creatinine (mg/dL)	9.74 ± 2.60	10.29 ± 3.11	9.75 ± 2.30	0.932
Uric acid (mg/dL)	6.18 ± 1.78	6.86 ± 1.68	6.35 ± 1.95	0.708
Na (meq/L)	138.12 ± 2.77	138.37 ± 3.82	138.11 ± 3.20	0.947
K (meq/L)	4.69 ± 0.79	4.56 ± 0.49	4.82 ± 0.84	0.321
Ca (mg/dL)	9.36 ± 0.66	9.31 ± 0.72	9.41 ± 0.92	0.718
P (mg/dL)	4.98 ± 1.42	5.03 ± 1.63	5.53 ± 1.64	0.043 *
C-reactive protein(mg/L)	6.00 (1.85–11.25)	3.30 (0.80–5.80)	4.20 (1.10–10.03)	0.212
Urea reduction rate	77 (71–79)	76 (65–81)	75.00 (71.25–79.00)	0.439
Kt/V (Daugirdes)	1.65 ± 0.27	1.56 ± 0.48	1.65 ± 0.33	0.888
nPCR (g/kg/day)	1.03 ± 0.24	1.07 ± 0.32	1.11 ± 0.66	0.381
TACurea	40.48 ± 13.03	47.46 ± 19.43	40.45 ± 11.91	0.803
Iron (μg/dL)	65 (50–90)	80 (51–98)	63.00 (49.00–88.75)	0.843
Ferritin (ng/mL)	465.0 (289.5–683.0)	349 (241–647)	386.50 (178.00–681.25)	0.150
TSAT (%)	34.04 ± 14.78	35.85 ± 10.19	33.82 ± 15.44	0.885
Cardiac/thoracic ratio	0.52 ± 0.05	0.52 ± 0.07	0.51 ± 0.06	0.234
i-PTH (pg/mL)	240 (114–707)	255.0 (80.9–777.0)	261 (125–701)	0.910
Ca × P product	46.82 ± 14.36	46.19 ± 14.19	52.52 ± 18.22	0.043 *

Notes: Data are presented as mean ± standard deviation or median (interquartile range). Abbreviations: RAAS: renin–angiotensin–aldosterone system; Abs, antibodies; WBC, white blood cell count; AST, aspartate transaminase; ALT, alanine transaminase; Alk-P, alkaline phosphatase; Bun, blood urea nitrogen; Kt/V, was used for the quantification of dialysis adequacy by the following formula: dialysis clearance of urea (K) multiplied by dialysis time (t), divided by the volume of distribution of urea (V); nPCR, normalized protein catabolic rate; TACurea, time average urea concentration; TSAT, transferrin saturation; i-PTH, intact parathyroid hormone *: statistically significant.

**Table 2:** Comparison of factors associated with dynamic changes of humoral responses after two doses of ChAdOx1.

	Group 1 (−/−), n = 68	Group 2 (−/+), n = 72	Group 3 (+/+), n = 22	*p* for Trend
Age, year	67.10 ± 12.69	64.07 ± 12.79	60.91 ± 15.00	0.040 *
Male, n (%)	35 (51.5)	40 (55.6)	14 (63.6)	0.602
Comorbidities, n (%)				
Diabetes	37 (54.4)	36 (50.0)	12 (54.50	0.844
Dyslipidemia	22 (32.4)	32 (44.4)	10 (45.5)	0.151
Liver cirrhosis	2 (3.0)	2 (2.8)	0 90)	0.518
Cerebrovascular disease	1 (1.5)	2 (2.8)	4 (18.2)	0.005 *
Congestive heart failure	16 (23.5)	6 (8.3)	1 (4.5)	0.006 *
Cardiovascular disease	23 (33.8)	24 (33.3)	8 (36.4)	0.882
Baseline medications, n (%)				
Immunosuppressant	4 (5.9)	5 (6.9)	0 (0)	0.474
RAAS blockade	34 (50.0)	27 (37.5)	11 (50)	0.559
β-blocker	33 (48.5)	21 (29.2)	12 (54.5)	0.601
Statins	20 (29.4)	30 (41.7)	10 (45.5)	0.098
Hemoglobin (g/dL)	9.98 ± 1.19	10.19 ± 1.18	10.22 ± 1.19	0.294
WBC (1000/μL)	6.10 (4.60–7.80)	5.90 (5.00–7.48)	6.35 (5.53–7.95)	0.272
Albumin (g/dL)	4.06 ± 0.38	4.01 ± 0.36	4.09 ± 0.32	0.918
Cholesterol (mg/dL)	157.91 ± 41.14	152.88 ± 33.28	147.45 ± 36.49	0.225
K (meq/L)	4.65 ± 072	4.88 ± 0.86	4.58 ± 0.73	0.704
Ca (mg/dL)	9.35 ± 0.67	9.34 ± 0.98	9.65 ± 0.63	0.253
P (mg/dL)	4.99 ± 1.46	5.57 ± 1.69	5.38 ± 1.48	0.103
C-reactive protein(mg/L)	4.25 (1.80–9.58)	3.30 (0.95–9.65)	6.20 (1.55–13.00)	0.880
Kt/V (Daugirdes)	1.62 ± 0.34	1.66 ± 0.32	1.58 ± 0.35	0.948
nPCR (g/kg/day)	0.99 (0.85–1.19)	1.02 (0.86–1.26)	1.06 (0.90–1.17)	0.731
TACurea	42.43 ± 15.26	40.79 ± 12.48	39.35 ± 10.39	0.310
Ferritin (ng/mL)	431.00 (262.50–658.25)	373. (182.0)	527.5 (117.0–623.0)	0.708
TSAT (%)	34.55 ± 13.60	32.59 ± 13.57	37.73 ± 20.18	0.693
Cardiothoracic ratio	0.52 ± 0.05	0.51 ± 0.06	0049 ± 0.06	0.071
i-PTH (pg/mL)	241.00 (101.88–720.50)	358.0 (144.5–792.0)	144.00 (97.28–523.00)	0.497
Ca × P product	46.64 ± 14.21	52.58 ± 18.86	52.31 ± 16.38	0.057

Notes: Data are presented as mean ± standard deviation and median (interquartile range). Abbreviations: NT_50_, SARS-CoV-2 50% neutralization titer; Group 1 (−/−), seronegative (NT_50_ < 35.13 IU/mL) from first dose to second dose; Group 2 (−/+), seronegative to first dose but changing to seropositive (NT_50_ ≥ 35.13 IU/mL) after second dose; Group 3 (+/+), seropositive from first dose to second dose; RAAS, renin–angiotensin–aldosterone system; Abs, antibodies; WBC, white blood cell count; AST, aspartate transaminase; ALT, alanine transaminase; Alk-P, alkaline phosphatase; Bun, blood urea nitrogen; Kt/V, was used for the quantification of dialysis adequacy by the following formula: dialysis clearance of urea (K) multiplied by dialysis time (t), divided by the volume of distribution of urea (V); nPCR, normalized protein catabolic rate; TACurea, time average urea concentration; TSAT, transferrin saturation; i-PTH, intact parathyroid hormone. *: statistically significant.

**Table 3:** β-coefficient between NT_50_ and independent variables.

	Simple Linear Regression		Multiple Regression Analysis, Model 1		Multiple Regression Analysis, Model 2	
	β ± SE	*p*	β ± SE	*p*	β ± SE	*p*
Age	−0.008 ± 0.004	0.081	-	-	−0.009 ± 0.005	0.079
Anti-S1 Abs ^$^	1.125 ± 0.209	<0.001 *	1.396 ± 0.052	<0.001 *	-	-
Anti-RBD Abs ^$^	1.448 ± 0.274	<0.001 *	-	-	-	-
Hemoglobin	0.062 ± 0.048	0.196	0.076 ± 0.032	0.018 *	-	-
MCV	0.005 ± 0.008	0.514	-	-	-	-
WBC ^$^	0.279 ± 0.366	0.448	-	-	-	-
Platelet	0.001 ± 0.001	0.145	-	-	-	-
Albumin	−0.092 ± 0.158	0.559	-	-	-	-
AST ^$^	0.122 ± 0.333	0.715	-	-	-	-
ALT ^$^	0.309 ± 0.253	0.224	0.287 ± 0.111	0.011 *	-	-
Alk-P ^$^	0.048 ± 0.216	0.823	-	-	-	-
Bilirubin ^$^	0.064 ± 0.388	0.870	-	-	-	-
Cholesterol	−0.002 ± 0.002	0.292	-	-	-	-
Triglyceride ^$^	−0.226 ± 0.228	0.323	-	-	-	-
Creatinine	−0.012 ± 0.023	0.588	-	-	−0.062 ± 0.029	0.034 *
Uric acid	0.002 ± 0.031	0.948	-	-	-	-
Na	0.005 ± 0.018	0.798	-	-	-	-
K	0.062 ± 0.072	0.388	-	-	-	-
Ca	0.050 ± 0.070	0.471	−0.062 ± 0.033	0.065	-	-
P	0.064 ± 0.036	0.078	-	-	-	-
C-reactive protein ^$^	−0.081 ± 0.098	0.409	−0.079 ± 0.041	0.055	-	-
URR ^$^	−0.215 ± 0.345	0.535	-	-	-	-
Kt/V	0.071 ± 0.171	0.678	-	-	-	-
nPCR	0.099 ± 0.108	0.361	-	-	-	-
TACurea	−0.004 ± 0.004	0.404	−0.006 ± 0.002	0.008 *	-	-
Ferritin ^$^	−0.224 ± 0.139	0.108	-	-	-	-
Iron ^$^	0.043 ± 0.320	0.894	-	-	-	-
TSAT	0.000 ± 0.004	0.919	-	-	-	-
intact-PTH	0.055 ± 0.111	0.624	-	-	-	-
Cardiac/thoracic ratio	−1.019 ± 1.058	0.337	-	-	-	-
Ca × P product	0.006 ± 0.003	0.057	0.005 ± 0.002	0.007 *	0.010 ± 0.004	0.021 *

Backward stepwise selection method was applied for multivariate analysis. Abbreviations: NT_50_, SARS-CoV-2 50% neutralization titer; Abs, antibodies; WBC, white blood cell count; AST, aspartate transaminase; ALT, alanine transaminase; Alk-P, alkaline phosphatase; Bun, blood urea nitrogen; Kt/V, was used for the quantification of dialysis adequacy by the following formula: dialysis clearance of urea (K) multiplied by dialysis time (t), divided by the volume of distribution of urea (V); nPCR, normalized protein catabolic rate; TACurea, time average urea concentration; TSAT, transferrin saturation. *: Statistically significant, ^$^: Log_10_ transformed. Model 1: Adjust for all variables; Model 2: Adjust for all variables, except anti-S1 and anti-RBD.

**Table 4:** Logistic regression analysis of factors associated with positive humoral response (NT_50_ > 35.13 IU/mL) to booster dose in first-dose seronegative HD patients.

	Univariate			Multivariable, Model 1			Multivariable, Model 2		
	OR	95% CI	*p* Value	OR	95% CI	*p* Value	OR	95% CI	*p* Value
Male	0.848	0.436–1.650	0.628	0.185	0.048–0.709	0.014 *	0.280	0.086–0.905	0.033
Age	0.981	0.955–1.008	0.162	0.983	0.946–1.023	0.405	-	-	-
Diabetes	1.194	0.614–2.319	0.602	0.662	0.248–1.766	0.410	-	-	-
Liver cirrhosis	0.929	0.127–6.785	0.942	6.4 × 10^7^	-	1	-	-	-
Cardiovascular disease	0.978	0.485–1.973	0.951	1.104	0.362–3.368	0.863	-	-	-
Immunosuppressants	0.838	0.215–3.259	0.798	0.684	0.098–4.768	0.701	-	-	-
RAAS blockade	0.600	0.306–1.177	0.137	1.381	0.528–3.614	0.511	-	-	-
β-blocker	5.194	2.641–10.215	<0.001 *	3.471	1.209–9.969	0.021 *	0.339	0.141–0.815	0.016
Hemoglobin	1.165	0.877–1.547	0.291	0.748	0.207–2.710	0.659	-	-	-
RBC	1.016	0.553–1.867	0.960	3.575	0.077–165.411	0.659	-	-	-
MCV	1.013	0.968–1.061	0.576	1.068	0.884–1.291	0.495	-	-	-
Albumin	0.674	0.270–1.683	0.399	0.189	0.032–1.115	0.066	0.276	0.067–1.134	0.074
ALT	1.005	0.981–1.030	0.691	1.041	0.991–1.094	0.113	-	-	-
K	1.444	0.939–2.223	0.095	1.749	0.811–3.770	0.154	1.915	1.054–3.476	0.033
C-reactive protein	1.004	0.985–1.023	0.714	0.989	0.964–1.015	0.411	-	-	-
Kt/V	1.506	0.544–4.156	0.432	20.295	2.486–165.683	0.005 *	7.469	1.283–43.484	0.025
nPCR	1.483	0.621–3.541	0.375	0.353	0.050–2.500	0.297	-	-	-
TSAT	0.989	0.965–1.014	0.398	0.983	0.952–1.016	0.309	-	-	-
Cardiothoracic ratio ^$^	0.155	0.000–112.604	0.579	0.050	0.000–222.929	0.485	-	-	-
Ca × P product	1.022	1.001–1.044	0.042*	1.022	0.986–1.060	0.232	-	-	-

Abbreviations: RBC, Red blood cell count; MCV, mean corpuscular volume; ALT, alanine transaminase; Kt/V, was used for the quantification of dialysis adequacy by the following formula: dialysis clearance of urea (K) multiplied by dialysis time (t), divided by the volume of distribution of urea (V); nPCR, normalized protein catabolic rate; TSAT, transferrin saturation. *: Statistically significant, ^$^: Log_10_ transformed. Model 1: All variables adjustment with enter method, Model 2: All variables adjustment with backward: Wald method.

**Table 5:** Humoral responses evolution of prime-booster vaccination of ChAdOx1 (Oxford–AstraZeneca).

	Hemodialysis Patients			Healthy Controls		
	First Vaccination (n = 174)	Second Vaccination (n = 162)	*p* Value	First Vaccination (n = 71)	Second Vaccination (n = 29)	*p* Value
Anti-S1 antibodies (EU/mL, GMT ± 95% CI)	2.75 (2.52–2.99)	7.82 (6.62–9.24)	<0.001 *	5.36 (4.50–6.39)	12.63 (7.83–20.36)	<0.001 *
Anti-RBD antibodies (EU/mL, GMT ± 95% CI)	2.43 (2.27–2.59)	5.41 (4.69–6.25)	<0.001 *	4.21 (3.61–4.91)	8.06 (5.27–12.31)	0.003 *
Predicted NT_50_ (median, IQR) (IU/mL)	6.85 (5.89–10.68)	109.72 (9.73–296.73)	<0.001 *	38.70 (9.45–186.71)	178.92 (33.04–544.67)	<0.001 *
Predicted NT_50_ (IU/mL, GMT ± 95% CI)	10.68 (9.09–12.54)	64.52 (49.79–83.60)		38.92 (27.26–55.57)	136.4 (74.96–248.4)	
Seroconversion, n (%)	23 (13.2)	94 (58.0)	<0.001 *	36 (50.7)	22 (75.9)	0.018 *
GMT ratio (fold, geometric mean ± 95% CI)	6.01 (4.72-7.66)			4.35 (2.14-8.82)		
Comparison of humoral response after first dose between HD and healthy contours (*p* value)	<0.001 *					
Comparison of humoral response after second dose between HD and healthy contours (*p* value)	0.037 *					
Comparison of GMT ratio between HD and healthy controls	0.135					

Notes: Data are presented as mean ± standard deviation and median (interquartile range). Abbreviations: NT50, SARS-CoV-2 50% neutralization titer; GMTs, geometric mean titers; GMT ratio, geometric mean fold increase in titers after the second dose vs. after the first dose. *: Statistically significant.

## Data Availability

Not applicable.

## Supplementary Materials

The following supporting information can be downloaded at: https://www.mdpi.com/article/10.3390/v14061149/s1, Table S1: Demographics and clinical characteristics comparisons between enrolled and non-enrolled patients on hemodialysis; Figure S1: Receiver operating characteristic curve illustrating the performance of Ca × P (AUC: 59%), Age (AUC: 43%), and the combination obtained by Ca × P dividing age (AUC: 60%) in predicting the development of NT50 over 35.13 IU/mL after prime-boost doses of ChAdOx1 vaccination.
