# Supplementary material for: Adequacy of Hemodialysis Serves as an Independent Predictor of Humoral Response to ChAdOx1 Prime-Boost Vaccination in Hemodialysis Patients
Source: Viruses. 2022 May 26;14(6):1149. doi: 10.3390/v14061149 (PMC9229890; doi:10.3390/v14061149)
Supplement: Supplementary file 1 [file viruses-14-01149-s001.zip › viruses-1738487-supplementary.pdf]

Table S1. Demographics and clinical characteristics comparisons between enrolled and non-enrolled patients on hemodialysis

	Enrolled (n= 162)	Non-enrolled (n= 336)	P value
Age, year	64.48 ± 13.06	66.58 ± 13.35	0.098
Male, n (%)	89 (54.9)	175 (52.1)	0.550
Hemoglobin (g/dL)	10.11 ± 1.19	10.04 ± 1.28	0.558
WBC (1000/μL)	6.53 ± 2.91	6.66 ± 2.85	0.637
Platelet (1000/μL)	192.59 ± 68.93	189.28 ± 68.34	0.616
Albumin (g/dL)	4.04 ± 0.36	3.98 ± 0.53	0.164
Cholesterol (mg/dL)	154.28 ± 37.18	153.39 ± 35.84	0.801
Triglyceride (mg/dL)	118.5 (80.00-175.75)	119 (81-184)	0.748
AST (U/L)	16.00 (13.00-20.75)	17 (13-22)	0.445
ALT (U/L)	14 (10-19)	15 (11-20)	0.074
Alk-P (U/L)	89.50 (70.75-125.00)	95 (72-139)	0.449
Total bilirubin (mg/dL)	0.4 (0.3-0.4)	0.3 (0.3-0.4)	0.615
Bun (mg/dL)	69.23 ± 21.44	71.33 ± 21.76	0.311
Creatinine (mg/dL)	9.81 ± 2.49	9.18 ± 2.76	0.014
Uric acid (mg/dL)	6.36 ± 1.87	6.46 ± 1.79	0.557
Na (meq/L)	138.14 ± 3.14	138.07 ± 3.24	0.805
K (meq/L)	4.75 ± 0.79	4.68 ± 0.84	0.368
Ca (mg/dL)	9.38 ± 0.83	9.29 ± 0.93	0.323
P (mg/dL)	5.30 ± 1.59	5.24 ± 1.54	0.661
C-reactive protein	4.25 (1.40-9.82)	3.50 (1.30-8.70)	0.449
Urea reduction rate	76 (71-79)	76 (71-80)	0.299
Kt/V (Daugirdes)	1.64 ± 0.34	1.68 ± 0.32	0.155
nPCR (g/kg/day)	1.08 ± 0.53	1.08 ± 0.53	0.978
TACurea	41.29 ± 13.43	42.18 ± 13.28	0.497
Iron (μg/dL)	65 (50-89)	65.00 (50.25-84.00)	0.737
Ferritin (ng/mL)	412.50 (220.25-667.00)	433.50 (191.25-665.50)	0.888
TSAT (%)	34.13 ± 14.65	32.40 ± 14.41	0.219
Cardiac/thoracic ratio	0.51 ± 0.06	0.52 ± 0.07	0.392
Ca x P product	50.05 ± 16.86	48.73 ± 15.04	0.380

Notes: Data are presented as mean ± standard deviation and median (interquartile range). Abbreviations: Abs, antibodies; WBC, white blood cell count; AST, aspartate transaminase; ALT, alanine transaminase; Alk-P, alkaline phosphatase; Bun, blood urea nitrogen; Kt/V, was used for the quantification of dialysis adequacy by the following formula: dialysis clearance of urea (K) multiplied by dialysis time (t), divided by the volume of distribution of urea (V); nPCR, normalized protein catabolic rate; TACurea, time average urea concentration; TSAT, transferrin saturation. *: statistically significant; $^{\$}$: Nonparametric, independent Sample Mann-Whitney U test.

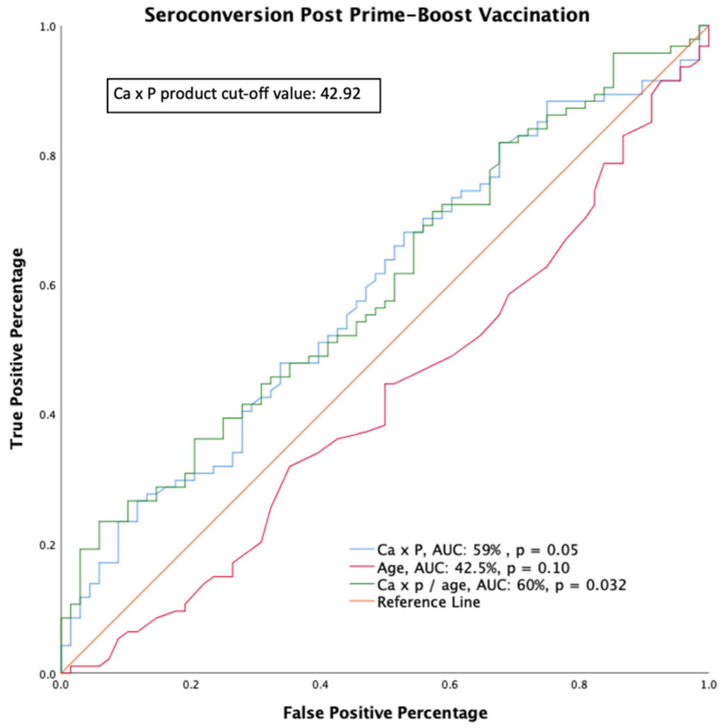

Figure S1. Receiver operating characteristic curve illustrating the performance of Ca × P (AUC: 59%), Age (AUC: 43%), and the combination obtained by Ca × P dividing age (AUC: 60%) in predicting the development of NT_{50} over 35.13 IU/mL after prime-boost doses of ChAdOx1 vaccination.
